# Biosynthesis Progress of High-Energy-Density Liquid Fuels Derived from Terpenes

**DOI:** 10.3390/microorganisms12040706

**Published:** 2024-03-30

**Authors:** Jiajia Liu, Man Lin, Penggang Han, Ge Yao, Hui Jiang

**Affiliations:** 1 State Key Laboratory of NBC Protection for Civilian, Beijing 102205, China; jiajialiu0802@163.com (J.L.);; 2 College of Biological Engineering, Sichuan University of Science and Engineering, Yibin 644005, China

**Keywords:** terpenes, high-energy-density liquid fuels, metabolic engineering, microbial cell factory, terpene synthases

## Abstract

High-energy-density liquid fuels (HED fuels) are essential for volume-limited aerospace vehicles and could serve as energetic additives for conventional fuels. Terpene-derived HED biofuel is an important research field for green fuel synthesis. The direct extraction of terpenes from natural plants is environmentally unfriendly and costly. Designing efficient synthetic pathways in microorganisms to achieve high yields of terpenes shows great potential for the application of terpene-derived fuels. This review provides an overview of the current research progress of terpene-derived HED fuels, surveying terpene fuel properties and the current status of biosynthesis. Additionally, we systematically summarize the engineering strategies for biosynthesizing terpenes, including mining and engineering terpene synthases, optimizing metabolic pathways and cell-level optimization, such as the subcellular localization of terpene synthesis and adaptive evolution. This article will be helpful in providing insight into better developing terpene-derived HED fuels.

## 1. Introduction

High-energy-density (HED) liquid fuel is a type of liquid propellant with higher density and volumetric net heat of combustion (NHOC) than conventional distilled fuel. It can be widely used in aerospace and national defense fields, such as spacecraft, rockets and missiles. HED fuel can provide more propulsion energy to improve aircraft range and speed and/or increase the payload. It can also effectively reduce the volume of fuel tanks, thereby making the aircraft smaller and more maneuverable [1,2,3]. Thus far, various HED fuels have been successfully synthesized and applied, obtained through polymerization, hydrogenation, isomerization and purification using petroleum-based raw materials.

For example, RJ-4 (RJ for ramjet) is the earliest synthesized polycyclic HED fuel with a density of 0.927 g/mL and a volumetric NHOC of 39.0 MJ/L, and is obtained from methylcyclopentadiene [1,4]. RJ-5 and RJ-7 are two currently reported HED fuels with densities exceeding 1 g/mL. RJ-5, with a density of 1.08 g/mL and a volumetric NHOC of 44.9 MJ/L, is synthesized from dicyclopentadiene [1]. RJ-7 is a mixed fuel with a density of 1.01 g/mL and a combustion value of 42.1 MJ/L [5]. However, their high freezing points limit further applications. JP-10, a high-density cruise missile fuel used extensively, is synthesized from dicyclopentadiene, with a density of 0.94 g/mL and a volumetric NHOC of 39.6 MJ/L. It can provide 13% more energy than kerosene (such as Jet A) [6]. The addition of HED fuels can increase missile range by 50% and improve carrying capacity by 17% without pollution [7,8]. In addition, high-tension cage hydrocarbons and nanoparticles can also be added to HED fuels to further improve fuel performance [9,10].

Although petroleum-based HED fuels have been widely used, the development of green synthesis of HED fuels has received widespread attention due to the shortage of petroleum resources and the environmental pollution crisis. The replacement of petroleum materials with biomass materials to achieve renewable and green fuels is an important research direction. Currently, the main sources of HED biofuels include terpenes and lignocellulose [3]. Among them, terpenes are favored due to their compact polycyclic structure and specific spatial configuration. Terpenes have unsaturated bonds, which can be modulated by direct hydrogenating or increasing the number of rings, providing more possibilities for the design of HED fuels with great application prospects [3]. Although terpenes are widely present in plants, their content is low, and they cannot be obtained in sufficient quantities for fuel applications through traditional extraction methods [11]. With the development of genetic engineering, systems biology and synthetic biology, researchers have discovered a production mode of synthesizing terpene-based fuels by constructing microbial cell factories to achieve green, low-cost and sustainable future manufacturing.

Some microorganisms have been metabolically engineered from the aspect of isoprenoid pathways for producing diverse terpenes, especially the two representative microbial hosts *Escherichia coli* and yeast. *E. coli* is the most widely studied prokaryotic microorganism model. It has a fast growth rate and can use a variety of substrates for aerobic or anaerobic growth. Moreover, diverse genetic manipulation tools and its clear genetic background have also contributed to *E. coli* acting as an important model microbial chassis for producing terpenes [12]. Yeasts, such as *Saccharomyces cerevisiae* and other non-conventional yeasts, including *Yarrowia lipolytica* and *Kluyveromyces lactis* et al., are single-celled eukaryotic microorganisms famous for their capabilities to ferment different sugars into targets. In terms of the ease and productivity of fermentation, yeasts exhibit an edge over bacterial strains since they have the ability to grow in more inexpensive media and have higher resistance to inhibitors [13]. This article focuses on the research progress of terpene-derived HED fuels, summarizing the properties of different types of terpene fuel molecules, the biosynthesis status of terpenes and the construction strategy of high-yield microbial cell factories for terpenes. Furthermore, it proposes feasible directions to obtain terpene fuels with excellent performance, as well as outlines the challenges facing their future development.

## 2. Properties of HED Fuels Derived from Terpenes

Terpenoids are compounds or derivatives formed by isoprene as the basic structure. According to the number of isoprene units (C5), terpenoids can be classified as hemiterpenoids (C5), monoterpenoids (C10), sesquiterpenoids (C15), diterpenoids (C20), etc. Terpenoids are widely present in plants or microorganisms and are the most widely reported natural compounds (more than 50,000 species) [14]. Some terpenes have been developed into fuels. Table 1 shows the actual properties of terpenes, including density, volumetric NHOC, etc. Monoterpenes and sesquiterpenes usually possess a high NHOC value and low freezing point, and therefore, they are often regarded as potential HED fuel molecules [15].

### 2.1. Monoterpene-Derived Fuels

Monoterpenes have two isoprene units and can be divided into linear or cyclic types, of which the cyclic type is more widely used. The main components of turpentine oil extracted from pine resin include α-pinene, β-pinene, and small amounts of camphene and limonene (with a density of 0.841–0.859 g/mL) [16]. Due to the compact structure of α-pinene and β-pinene, the hydrogenated products catalyzed with Ni-SiO_2_ and Pd-Al_2_O_3_ (the yield of products was over 95%) could be used as HED fuel with a density of 0.86 g/mL, a kinematic viscosity of 13 mm^2^/s at −40 °C and a freezing point below −75 °C [17]. Harvey et al. used Nafion acid to catalyze the dimerization of β-pinene with a yield of 90%. The resulting pinene dimers were directly hydrogenated to produce a better HED fuel with a density of 0.938 g/mL and a volumetric NHOC of 39.5 MJ/L, which is similar to JP-10 [16]. The α-pinene dimer and camphene dimer showed similar performance, including their density, volumetric NHOC, viscosity and freezing point, since during the polycondensation of α-pinene it preferentially forms camphene rather than dimers [16].

Limonene also has good properties, especially low-temperature performance. It was reported that mixing diesel fuel with 10% hydrogenated limonene can effectively reduce the cloud point and viscosity of the blended fuel, indicating that limonene can be used as a substitute for traditional fuels without changing the engine [18]. The volumetric NHOC of limonene was close to JP-10, reaching 39.15 MJ/L, while the density of limonene was lower than that of JP-10 [19]. The dimerization of limonene could improve the density to 0.914 g/mL; however, due to the lack of a compact structure, the density of the limonene dimer was inferior to that of the pinene or camphene dimers [20]. In addition to pure pinene, limonene and camphene, crude turpentine was also dimerized with catalysts, including Nafion SAC-13, MMT-K10 and HPW/MCM-41, to generate a high-density turpentine dimer fuel (TDF), of which the product’s composition was similar to that of a pure pinene dimer: the density was up to 0.93 g/mL and the volumetric NHOC value was close to JP-10 [16,20,21]. The above research clearly shows that the direct dimerization of monoterpenes or crude turpentine to generate HED fuels can take full advantage of the monomers. Although the combustion value and density of dimer fuels are very high, the viscosity is too high to be applied at a low temperature [21]. To solve this problem, the dimer HED fuel was mixed with other low-viscosity components, including JP-8 and JP-10, resulting in mixed fuels with a superior combustion value and low-temperature performance [17,21].

Cyclopropanation has been widely studied due to its significant impact on fuel density and heating value. As early as the Soviet era, a cyclopropanation fuel named Syntin had been used in rocket engines, which could effectively increase the payload [22]. In 2022, Keasling et al. synthesized a class of polycyclopropanated fatty acid methyl ester fuels (POP-FAMEs), the densities and volumetric NHOC values of which exceeded those of JP-10 [23]. The volumetric energy of terpenes could also be improved further through cyclopropanation. Lebel et al. discovered that the density and volumetric NHOC of the cyclopropanated products of limonene and turpentine were improved by 3–4% [24]. In addition, Liu et al. effectively cyclopropanated the monoterpene myrcene with a conversion rate of 69.8%; the resulting product had a density of 0.85 g/cm^3^, a volumetric NHOC of 36.89 MJ/L, a freezing point below −70 °C and a specific impulse higher than that of RP-1 and JP-10 [25]. Furthermore, monoterpenes including sabinene, 3-carene, α-pinene, β-pinene and limonene were also cyclopropanated; the density and volumetric NHOC of the resulting fuels were significantly improved, and they showed a kinematic viscosity of 8.3 mm^2^/s at −20 °C, exhibiting great application prospects [26,27].

Oxygen-containing monoterpenes are also an important source of HED fuels. 1,8-cineole from Eucalyptus is an intermediate of the aviation fuel AMJ-700t (10% cymene, 50% limonene and 40% farnesene) [28]. Meylemans et al. used Amberlyst-15, Nafion SAC-13 and Montmorillonite K10 to catalyze 1,4-cineole and 1,8-cineole into HED fuels, with the conversion rate of 1,4-cineole reaching 100% using Amberlyst-15 [29]. Yang et al. reported a new catalytic method, biTCP, to efficiently catalyze 1,8-cineole into p-menthane with a 99% conversion rate [15]. Linalool, an acyclic monoterpene, is an important fragrance component of lavender, rose and other plants, which can be used to synthesize the traditional HED fuel RJ-4 [30]. Harvey et al. used linalool as a raw material to obtain a mixed fuel composed of p-menthane and 2,6-dimethyloctane (DMO) through a one-pot reaction, which had a higher heating value and density than the jet fuel Jet-A [31].

### 2.2. Sesquiterpene-Derived Fuels

Sesquiterpenes are more complex in structure than monoterpenes and have also received widespread attention in fuel applications. The linear sesquiterpene farnesene has a lower density and NHOC than its hydrogenated product farnesane, which is not suitable for use as a HED fuel alone but can be used as a jet fuel [32]. Pamela et al. found that the volumetric NHOC and density of the monocyclic sesquiterpene bisabolene were very similar to D2 diesel and could be used as a substitute [33]. Harvey et al. tested the properties of the bicyclic sesquiterpenes, including valencene, caryophyllene and premnaspirodiene, and the results showed that the hydrogenated fuels of the three compounds had a high volumetric NHOC (37.01–37.78 MJ/L) but lower density [34]. Subsequently, caryophyllene was catalyzed into mixtures of isomers with Nafion SAC-13, and the mixture fuel had a density of 0.85–0.9 g/mL and a volumetric NHOC of 37.07–39.30 MJ/L [34]. They also mixed the caryophyllene isomers with a hexene dimer at a 40:60 ratio to obtain blended fuel with a density of 0.806 g/mL, a volumetric NHOC of 34.73 MJ/L and a kinematic viscosity of 8.3 mm^2^/s at −20 °C, which are close to JP-5 [35].

Cedarwood oil (CWO) is a mixture of sesquiterpenes, mainly composed of tricyclic sesquiterpenes, including thujopsene, α-cedrene, β-cedrene and cedrol [36]. Harrison et al. hydrogenated CWO containing 32% α-cedrene, 7% β-cedrene, 51% thujopsene and some other minor sesquiterpenes and obtained HCWO fuels with a density of 0.917 g/mL and a volumetric NHOC of 39.13 MJ/L, which are similar to JP-10. However, the obtained fuels had a high viscosity, with a kinematic viscosity of 54 mm^2^/s at −20 °C, which is higher than that of JP-8 (<8.0 mm^2^/s) or JP-10, and a kinematic viscosity of 6.2 mm^2^/s at 40 °C, which is higher than that of traditional diesel (2.1–4.1 mm^2^/s) [37]. Furthermore, by catalyzing cedrol to generate cedrane, which possesses better properties, including higher density, a higher volumetric NHOC value and lower viscosity (with kinematic viscosity 33 mm^2^/s at −20 °C and 4.8 mm^2^/s at 40 °C) than that of HCWO, it exhibited great potential for practical applications [37].

Moreover, some studies have investigated the properties of sesquiterpene-derived fuels using theoretical calculations. It was reported that the theoretical predicted gravity NHOC values of three tricyclic sesquiterpene hydrogenation products, epi-isozizane, pentalenane and α-isocomane, were 42.584 MJ/kg, 42.609 MJ/kg and 42.783 MJ/kg, respectively, which are close to the commercial jet fuel Jet-A1 [38]. Geiselman et al. also found that the volumetric NHOC of saturated prespatane was 41.46 MJ/L, which is better than that of JP-10 [39]. Butcher et al. analyzed 33 compounds from fungi and discovered that five sesquiterpenes, longifolene, β-cubebene, α-ylangene, β-patchoulol and α-santalene, have great potential to be applied for HED fuels [40]. Recently, our research team systematically calculated the combustion properties of 122 sesquiterpene skeletal compounds already reported and found that many hydrogenated products of these molecules have superior properties to JP-10 in terms of their volumetric NHOC value, density and other characteristics. Subsequently, the actual combustion performance of pentalenene and presilphiperfol-1-ene was tested, and the results showed that the hydrogenated pentalenane and presilphiperfol-1-ane have volumetric NHOC values of 40.55 MJ/L and 39.24 MJ/L, respectively, which were close to the theoretical predicted values [32].

**Table 1:** The properties of terpene-derived HED fuels.

Fuel	Structure	NHOC/(MJ/L)	Density/(g/mL)	Viscosity/mm^2^/s	Freezing Point/°C	Ref.
β-Pinene	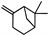	36.89	0.86 (20 °C)	—	—	[16]
α-Pinene	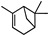	36.89	0.86 (20 °C)	—	—	[16]
α-Pinane	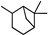	37.08	0.86 (15 °C)	11.23 (−40 °C)	<−75	[21]
Limonene	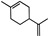	39.15	~0.87 (20 °C)	2.35 (−20 °C)	−74	[19]
Sabinane	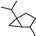	35.05	0.81 (15 °C)	4.809 (−40 °C)	—	[41]
Myrcene	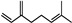	34.32	0.79 (20 °C)	—	—	[27]
p-Menthane	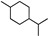	34.72	0.804 (15 °C)	5.19 (−40 °C)	<−70	[22,42]
Hydrogenated β-pinene dimer	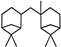	39.50	0.938 (20 °C)	35.05 (40 °C)	−30	[16]
α-Pinene dimer	-	39.31	0.935 (20 °C)	34.68 (40 °C)	−52	[16]
Camphene dimer	-	39.58	0.941 (20 °C)	34.96 (40 °C)	−54	[16]
Limonene dimer	-	38.36	0.914 (20 °C)	25.86 (40 °C)	−78	[16]
Farnesane	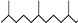	33.93	0.77 (20 °C)	2.35 (40 °C)	−52	[32]
Pentalenene	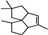	40.73	0.91 (20 °C)	—	−63.5	[32]
Pentalenane	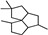	40.55	0.89 (20 °C)	—	<−67	[32]
Pentalenene cyclopropanation	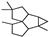	38.43	0.90 (20 °C)	—	<−53	[32]
Presilphiperfol-1-ane	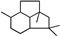	39.24	0.92 (20 °C)	—	−47	[32]
Hydrogenated cedarwood oil	-	39.25	0.92 (20 °C)	6.2 (40 °C)	<−80	[37]
Valencane	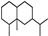	37.73	0.88 (20 °C)	50.2 (−20 °C)	—	[34]
Premnaspirodiane	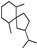	37.78	0.88 (20 °C)	42.9 (−20 °C)	—	[34]
Caryophyllane	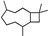	37.01	0.85 (20 °C)	60.5 (−20 °C)	—	[34]
Cedrane	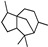	39.47	0.923 (20 °C)	33 (−20 °C)	—	[37]

## 3. Microbial Synthesis of Monoterpene and Sesquiterpene Fuels

Monoterpenes and sesquiterpenes naturally exist in plants, but their contents are extremely low. Therefore, it is difficult to achieve large-scale production for fuel applications through direct extraction and isolation methods. In addition, slow plant growth and geographical limitations further restrict the extraction of terpenes from plant sources [43]. The microbial synthesis of terpenes provides an efficient and scalable alternative platform for the production of monoterpene and sesquiterpene fuels. By constructing biosynthetic pathways of the target production in microbial cells, specific synthesis and renewable production of the desired products can be achieved effectively.

**Figure 1:**
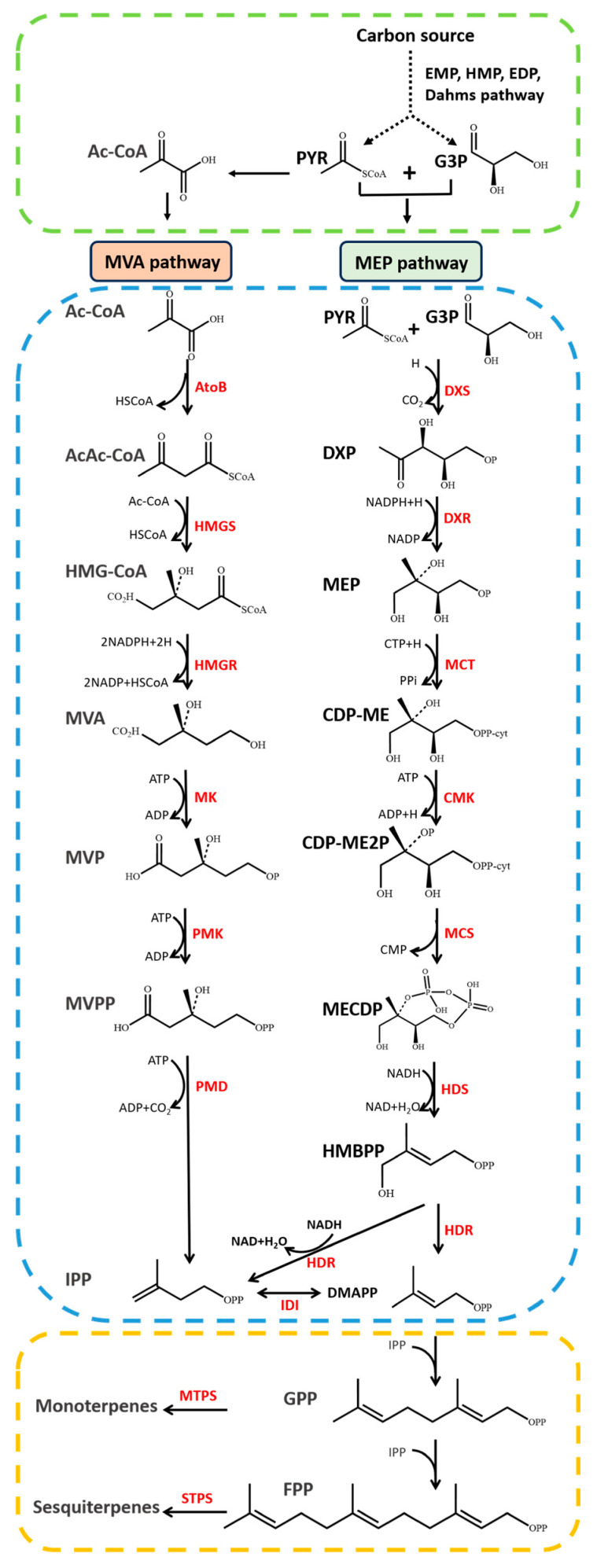
Monoterpene and sesquiterpene biosynthesis pathway. Abbreviations are shown in Abbreviations section.

The biosynthesis of monoterpenes and sesquiterpenes is based on isopentenyl diphosphate (IPP) and dimethylallyl diphosphate (DMAPP). In microorganisms, IPP and DMAPP are usually synthesized through two pathways, the mevalonate (MVA) pathway or the 2-C-methyl-D-erythritol 4-phosphate (MEP) pathway (Figure 1). The MVA pathway exists in fungi, bacteria, the plant cytoplasm and animal cells. It starts with acetyl-CoA and undergoes catalytic reactions to form acetoacetyl-CoA, HMG-CoA and mevalonic acid, ultimately generating the precursors IPP and DMAPP [44]. The MEP pathway mainly exists in bacteria, algae and plant chloroplasts. It starts with pyruvic acid and glyceraldehyde-3-phosphate and undergoes catalytic reactions to form 1-deoxy-D-xylulose-5-phosphate (DXP) and MEP and eventually produces IPP, which is isomerized into DMAPP by isopentenyl diphosphate isomerase [45]. DMAPP and IPP condense to form the precursor geranyl pyrophosphate (GPP) for monoterpene synthesis catalyzed by monoterpene synthase (MTPS). GPP and IPP further condense to form the precursor farnesyl pyrophosphate (FPP) for sesquiterpene synthesis catalyzed by sesquiterpene synthase (STPS) [44]. Some monoterpenes and sesquiterpenes have been successfully synthesized in microorganisms, as shown in Table 2.

### 3.1. Microbial Synthesis of Monoterpenes

Compared to sesquiterpenes, the titers of monoterpene compounds are relatively lower. Rolf et al. optimized the fermentation conditions to achieve high limonene production using glycerol as the carbon source in *E. coli*, and the final limonene yield reached 3.6 g/L in the fermenter with a rate of 0.15 g/L/h, which is the highest yield reported [45]. Tashiro et al. screened efficient variants of pinene synthase to achieve the efficient synthesis of pinene. By co-expressing the variant with MVA-pathway-related genes and isopentenyl diphosphate isomerase in *E. coli*, the pinene production reached 140 mg/L in shake-flask fermentation [46]. In addition, researchers also synthesized pinene through whole-cell biocatalysis. A titer of 166.5 mg/L pinene was achieved by co-cultivating *E. coli* expressing the MVA pathway with the *E. coli* expressing GPP synthase and pinene synthase [47]. Our research group developed a strategy based on quorum sensing and the dynamic regulation of small RNAs to improve pinene synthesis in *E. coli*. By dynamically inhibiting the expression of multiple genes involved in competing pathways for pinene synthesis without affecting cell growth, the pinene yield reached 165.3 mg/L in shake-flask fermentation, reaching an increase of 365.3% compared to the control strain [48]. Additionally, sabinene has also been successfully synthesized in *E. coli*. Zhang et al. introduced the sabinene synthase from *Salvia pomifera* and the GPP synthase from *Abies grandis* into *E. coli*, achieving a sabinene yield of 82.18 mg/L in shake-flask fermentation and 2.65 g/L in the fermenter [49].

Furthermore, the synthesis of oxygen-containing monoterpenes has also received extensive attention. 1,8-cineole was synthesized in *Rhodosporidium toruloides* with a lignocellulosic hydrolysate medium and reached a titer of 1.4 g/L [50]. Hoshino et al. discovered that the (S)-linalool synthase (AaLINS) from *Actinidia arguta* and the (R)-linalool synthase (ScLINS) from *Streptomyces clavuligerus* exhibited the highest efficiency among 16 linalool synthases. When AaLINS and ScLINS were expressed simultaneously with a mutated FPP synthase (ispA*) in *E. coli*, the yields of (S)-linalool and (R)-linalool reached 5.6 g/L and 3.71 g/L, respectively, which are the highest reported yields so far [51].

### 3.2. Microbial Synthesis of Sesquiterpenes

The microbial synthesis research of sesquiterpenes is more extensive, and many sesquiterpene compounds have been synthesized (Table 2). The synthesis of β-farnesene has been achieved via commercial application by Amyris Inc., achieving a fermentation yield of 130 g/L in a 2000 L fermenter in *S. cerevisiae* [52]. They introduced four non-native metabolic reactions into *S. cerevisiae* to rewire the central carbon metabolism, enabling the biosynthesis of cytosolic acetyl-CoA with a reduced ATP requirement and loss of carbon to CO_2_-emitting reactions and improved the pathway redox balance [52]. These changes dramatically increased the productivity in industrial fermentations that are oxygen-constrained and provided a viable strategy for the large-scale production of other terpenes in *S. cerevisiae*. In addition, other researchers have also synthesized β-farnesene using *Y. lipolytica* as the host cell. Bi et al. achieved a fermentation titer of 28.9 g/L β-farnesene in *Y. lipolytica* by modulating the regeneration of NAD(P)H, the supply of precursors and ATP [53]. Liu et al. achieved a high yield of β-farnesene (31.9 g/L) using waste edible oil as a carbon source by genetically engineering the lipid synthesis pathway and combining β-farnesene synthase engineering [54]. Apart from β-farnesene, the microbial synthesis of α-farnesene has also shown good progress. The highest titer of α-farnesene was achieved in *S. cerevisiae*, reaching 38.8 g/L [55].

Monocyclic sesquiterpenes such as bisabolene, germacrene and humulene have also been efficiently synthesized in microorganisms. The microbial synthesis of α-bisabolene was achieved in *Y. lipolytica* by positioning the α-bisabolene synthetic pathway into peroxisomes and increasing the supply of ATP, achieving a yield of 15.5 g/L [55]. Recently, a new γ-bisabolene synthase, AcTPS5, was characterized from *Acremonium chrysogenum*, and the highest titer of γ-bisabolene, which reached 2.69 g/L, was achieved by expressing AcTPS5 both in the cytosol and peroxisomes of *S. cerevisiae* [56]. The production of germacrene A reached 39 g/L by screened germacrene A synthase and increased the synthesis of acetyl-CoA by reconstructing the endogenous MVA pathway [57]. Our research group identified a new germacrene D synthase, AcTPS1, and by increasing the copy number of the limited enzyme of the MVA pathway and *Actps1*, together with inhibiting the expression of the competing pathway, the efficient synthesis of germacrene D was realized in *S. cerevisiae*, achieving a titer of 7.9 g/L [58]. Huang et al. identified a bicyclogermacrene synthase in *Penicillium expansum* and realized the synthesis of bicyclogermacrene in *E. coli* with a titer of 188 mg/L [59].

In addition, Deng et al. achieved the efficient synthesis of bicyclic sesquiterpenes including valencene and eremophilene in *S. cerevisiae* through screening and engineering synthases and optimizing the synthetic pathway, achieving titers of 16.6 g/L and 34.6 g/L, respectively [60]. Recently, our research group synthesized 17 sesquiterpenes with high productions in *S. cerevisiae*, such as pentalenene, α-santalene, presilphiperfol-1-ene and protoilludane, and all achieved titers exceeding 10 g/L [32]. With the discovery of more sesquiterpene synthases, more and more tricyclic sesquiterpenes, including oxygenated sesquiterpenes, have been successfully synthesized by microorganisms, including longifolene, zizaene, viridiflorol and patchoulol. Shukal et al. improved the translation efficiency of viridiflorol synthase and combined strain modification, achieving a titer of 25.7 g/L viridiflorol in *E. coli* [61]. The efficient synthesis of sesquiterpenes is crucial for their application in fuel development.

The above description shows that remarkable progress has been made in the production of terpene fuels through microbial fermentation using glucose, sucrose or glycerol as the carbon source (Table 2). By using a chassis that can efficiently utilize other types of carbon sources, such as *R. toruloides* [50], *Trichoderma atroviride* [62] and endophytic fungus *Annulohypoxylon* [63], terpene fuels can be produced from non-food biomass like cellulose. Additionally, algae, with their photosynthetic ability and faster growth compared to plants, have gained more attention regarding terpene fuel production [64]. Algae can also grow and reproduce in wastewater or seawater, effectively utilizing nitrogen and phosphorus sources in wastewater, leading to potential applications in bioremediation and reducing freshwater dependency during fermentation [65,66]. Lin et al. achieved the efficient synthesis of monocyclic limonene in cyanobacteria by regulating the expression of GPP synthase and other synthases involving the MEP pathway, resulting in a limonene titer of 16.4 mg/L after two days of cultivation [67]. However, the production of pinene in algae is significantly lower compared to limonene. Umeno et al. and Chauvat et al. separately synthesized pinene in *Synechocystis* sp. PCC 6803, achieving a titer of 40 μg/L at 168 h and 80 μg/L at 21 days, respectively [46,68]. Researchers have also achieved the synthesis of sesquiterpene in algae. Li et al. achieved the synthesis of β-caryophyllene by constructing the β-caryophyllene synthetic pathway and enhancing the precursor supply in *Synechocystis* UTEX 2973, and the production of β-caryophyllene in a photobioreactor reached 212.37 μg/L with high-density cultivation [69]. These studies have laid a solid foundation for the future synthesis of HED fuels using non-food biomass.

**Table 2:** Summary of microbial synthesis of monoterpenes and sesquiterpenes.

Product	Chassis	Synthetase	Conditions	Carbon Source	Production	Ref.
**Monoterpene**						
Limonene	*E. coli* BL21 (DE3)	Limonene synthase from *Mentha spicata*	Fed-batch fermentation/3.1 L	Glycerol	24 h, 3.63 g/L	[45]
α-Pinene	*E. coli* BW25113	PS from *Pinus taeda*	Whole-cell biocatalysis/50 mL	Glucose	28 h, 166.50 mg/L	[47]
	*E. coli* MG1655	PS from *Abies grandis*	Shake-flask fermentation/10 mL	Glucose	48 h, 165.10 mg/L	[48]
Sabinene	*E. coli* BL21(DE3)	SabS1 from *Salvia pomifera*	Fed-batch fermentation/5 L	Glycerol	24 h, 2.65 g/L	[49]
Myrcene	*E. coli* MG1655	CsMS from *Cannabis sativa*	Shake-flask fermentation/10 mL	Glucose	72 h, 510.38 mg/L	[70]
γ-Terpinene	*E. coli* BL21 (DE3)	γ-Terpinene synthase *Thymus vulgaris*	Fed-batch fermentation/2 L	Glycerol	72 h, 275.41 mg/L	[71]
1,8-Cineole	*R. toruloides* IFO0880	HYP3 from *Hypoxylon* sp. E7406B	Fed-batch fermentation/2 L	Corn stover hydrolysate	168 h, 1.4 g/L	[50]
Geraniol	*E. coli* DH5α	GES from *Valeriana officinalis*	Shake-flask fermentation	Glycerol	48 h, 2.12 g/L	[72]
	*S. cerevisiae* CEN.PK102-5B	geraniol synthase from *Valeriana officinalis*	Fed-batch fermentation/1 L	Glucose	120 h, 1.69 g/L	[73]
(S)-Linalool	*P. ananatis*	AaLINS from *Actinidia arguta*	Test tube/5 mL	Glucose	24 h, 5.6 g/L	[51]
(R)-Linalool	*P. ananatis*	ScLINS from *Streptomyces clavuligerus*	Test tube/5 mL	Glucose	24 h, 3.71 g/L	[51]
Nerolidol	*S. cerevisiae* CEN.PK2-1C	NES from *Actinidia chinensis*	Shake-flask fermentation/50 mL	Glucose	72 h, 4.2 g/L	[74]
**Sesquiterpene**						
β-Farnesene	*S. cerevisiae* CEN.PK2-1C	FS from *Artemisia annua*	Industrial bioreactors/200,000 L	Glucose and sucrose	2.24 g/L/h, 130 g/L	[52]
	*Y. lipolytica* Po1f	FS from *A. annua*	Fed-batch fermentation/2 L	Glucose	240 h, 28.9 g/L	[53]
	*Y. lipolytica* Po1f	FS from *A. annua*	Fed-batch fermentation/5 L	Waste cooking oil	216 h, 35.2 g/L	[54]
	*Y. lipolytica* MYA-2613	FS from *A. annua*	Fed-batch fermentation/2 L	Glucose	175 h, 22.8 g/L	[75]
α-Farnesene	*S. cerevisiae* CEN.PK2-1D	AFS from *Malus domestica*	Fed-batch fermentation/15 L	Glucose and sucrose	120 h, 38.8 g/L	[32]
	*S. cerevisiae* CEN.PK113-5D	CsAFS from *Camellia sinensis*	Fed-batch fermentation/5 L	Glucose and sucrose	145 h, 28.3 g/L	[76]
	*Y. lipolytica* Po1f	FS from apple seeds	Fed-batch fermentation/1 L	Glucose	288 h, 25.5 g/L	[77]
Pentalenene	*S. cerevisiae* CEN.PK2-1D	PentS from *Streptomyces exfoliatus*	Fed-batch fermentation/15 L	Glucose and sucrose	120 h, 10.8 g/L	[32]
Presilphiperfol-1-ene	*S. cerevisiae* CEN.PK2-1D	Cgl06493-COP from *Colletotrichum gloeosporioides*	Fed-batch fermentation/15 L	Glucose and sucrose	120 h, 22.7 g/L	[32]
β-Copaene	*S. cerevisiae* CEN.PK2-1D	Copu2 from *Coniophora puteana*	Fed-batch fermentation/15 L	Glucose and sucrose	120 h, 6.8 g/L	[32]
Epi-isozizaene	*S. cerevisiae* CEN.PK2-1D	SCO5222 from *Streptomyces*	Fed-batch fermentation/15 L	Glucose and sucrose	120 h, 4.7 g/L	[32]
Thujopsene	*S. cerevisiae* CEN.PK2-1D	BarS from *Arabidopsis thaliana*	Fed-batch fermentation/15 L	Glucose and sucrose	120 h, 1.2 g/L	[32]
α-Barbatene	*S. cerevisiae* CEN.PK2-1D	BarS from *Arabidopsis thaliana*	Fed-batch fermentation/15 L	Glucose and sucrose	120 h, 1.6 g/L	[32]
Protoilludene	*S. cerevisiae* CEN.PK2-1D	OMP7 from *Omphalotus olearius*	Fed-batch fermentation/15 L	Glucose and sucrose	120 h, 12.1 g/L	[32]
α-Bisabolene	*Y. lipolytica* Po1g	BIS from *Abies grandis*	Fed-batch fermentation/5 L	Waste cooking oil	144 h, 15.5 g/L	[55]
β-Bisabolene	*Y. lipolytica* Po1g	BS from *Zingiber officinale*	Shake-flask fermentation/25 mL	Glucose	120 h, 68.2 mg/L	[78]
γ-Bisabolene	*S. cerevisiae* CEN.PK2-1D	AcTPS5 from *Acremonium chrysogenum*	Fed-batch fermentation/5 L	Glucose and ethanol	34.7 h, 2.69 g/L	[56]
Germacrene A	*Y. lipolytica* W29	dlGAS from *Daldinia loculata*	Fed-batch fermentation/5 L	Glucose	240 h, 39 g/L	[57]
(-)-Germacrene D	*S. cerevisiae* CEN.PK2-1D	AcTPS1 from *A. chrysogenum*	Fed-batch fermentation/5 L	Glucose and ethanol	94 h, 7.9 g/L	[58]
(+)-Bicyclogermacrene	*E. coli* BL21(DE3)	PeTS4 from *Penicillium expansum*	Shake-flask fermentation/50 mL	Glucose	72 h, 188 mg/L	[59]
Valencene	*S. cerevisiae* CEN.PK2-1D	EgVS from *Eryngium* *glaciale*	Fed-batch fermentation/15 L	Glucose and sucrose	120 h, 16.6 g/L	[79]
Premnaspirodiene	*S. cerevisiae* BY4741	HPS from *Hyoscyamus muticus*	Shake-flask fermentation/30 mL	Glucose	144 h, 170.23 mg/L	[80]
Caryophyllene	*E. coli* BL21(DE3)	TPS7 from *Nicotiana tabacum*	Fed-batch fermentation/5 L	Glucose	72 h, 5.14 g/L	[81]
α-Humulene	*Y. lipolytica* Po1f	ACHS2 from *Aquilaria crassna*	Fed-batch fermentation/5 L	Glucose	40 h, 21.7 g/L	[82]
δ-Cadinene	*S. cerevisiae* CEN.PK2-1C	LsSqTPS2 from *Leonurus sibiricus L.*	Shake-flask fermentation/50 mL	Glucose	120 h, 76.23 mg/L	[83]
Longifolene	*S. cerevisiae* CEN.PK113-5D	psTPS from *Pinus sylvestris*	Fed-batch fermentation	Glucose	180 h, 1.24 g/L	[84]
α-Santalene	*K. phaffii*	SAS	Fed-batch fermentation	Glucose	90 h, 21.5	[85]
α-Isocomene	*E. coli* DH1	MrTPS2 from *Matricaria recutita*	Shake-flask fermentation	Glucose	72 h, 77.5 mg/L	[38]
(-)-Eremophilene	*S. cerevisiae* CEN.PK2-1D	OsaTPS07 from *Ocimum sanctum*	Fed-batch fermentation/5 L	Glucose and ethanol	90 h, 34.6 g/L	[60]
Zizaene	*E. coli* BL21(DE3)	ZS from *Chrysopogon zizanioides*	Fed-batch fermentation/2 L	Glucose	72 h, 211.13 mg/L	[86]
Viridiflorol	*E. coli* MG1655	VS from *Agrocybe aegerita*	Fed-batch fermentation/250 ml	Glucose	60 h, 25.7 g/L	[61]
Patchoulol	*Y. lipolytica* Po1f	Patchoulol synthase from *Pogostemon cablin*	Fed-batch fermentation/5 L	Glucose	180 h, 2.86 g/L	[87]
τ-Cadinol	*E. coli* BL21(DE3)	τ-Cadinol synthase from *Lavandula angustifolia*	Fed-batch fermentation/5 L	Glucose	168 h, 15.2 g/L	[88]
Nerolidol	*S. cerevisiae* CEN.PK2-1C	NES from *Actinidia chinensis*	Shake-flask fermentation/50 mL	Glucose	72 h, 4.2 g/L	[74]

## 4. Strategies for Constructing Efficient Microbial Cell Factories for Terpenes

The construction of efficient microbial cell factories is crucial for the green manufacturing of terpene fuels. The exogenous terpene synthesis pathway utilizes the precursors, energy and co-factors in the host to synthesize target products. However, the inefficient expression of the exogenous genes and the inhibition of the growth and metabolism of the host caused by exogenous pathways could severely limit the synthesis of a desired product. Therefore, the rational design, modification and reconstruction of microbial metabolic pathways are key issues in the industrial-scale production of terpene-derived fuels. This section focuses on the mining and engineering of terpene synthases, optimizing the metabolic pathways and redesigning chassis cells, providing an overview of the strategies for constructing efficient microbial cell factories for terpenes synthesis.

### 4.1. Terpene Synthase Mining and Engineering

#### 4.1.1. Mining of Terpene Synthases

**Figure 2:**
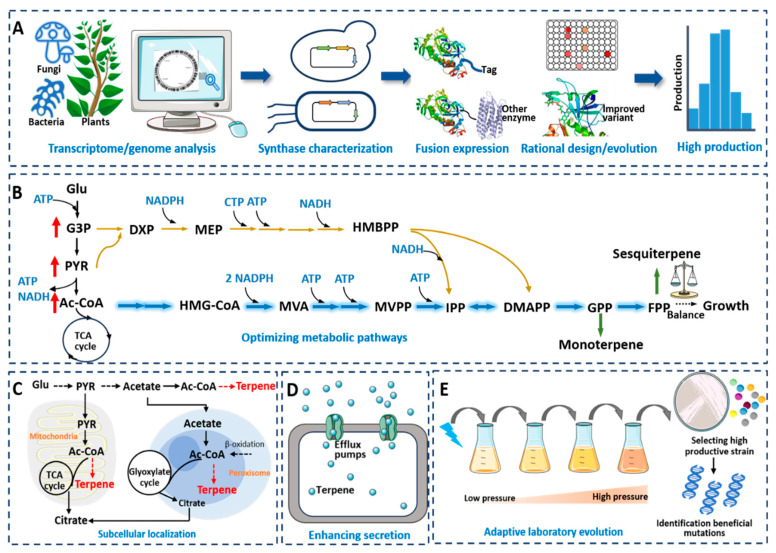
(**A**) Mining and engineering of terpene synthases for overexpression of terpenes. (**B**) Optimization of terpene synthetic pathways and upstream or downstream pathways to improve terpene production. (**C**) Subcellular localization of mitochondria and peroxisome for terpene synthesis. (**D**) Increasing cellular tolerance by overexpressing efflux pumps. (**E**) Adaptive laboratory evolution for improving the terpene production.

In microbial cells, the synthesis of monoterpenes and sesquiterpenes relies on exogenously introduced terpene synthases to catalyze the formation of the corresponding targets from precursor GPP/FPP. Therefore, the activity and selectivity of terpene synthases play a crucial role in the efficiency and purity of terpene synthesis. For terpenes that have not been successfully synthesized in microbes or have low production yields, mining the corresponding synthases is the first step in achieving their efficient synthesis (Figure 2A). Effective ways to obtain new or better terpene synthases include database mining and the exploration of microbial or plant genomes/transcriptomes [89]. Takano et al. expressed 22 terpene synthases derived from bacteria in *E. coli*, and 15 of them were found to be active in generating corresponding products, including monoterpenes, sesquiterpenes and diterpenes, with some showing high activity, which was conducive to the subsequent large-scale synthesis of terpenes in microbes [90]. Recently, our research group discovered a (+)-cubenene synthase in *A. chrysogenum*, which was the first report of a (+)-cubenene synthase, ultimately achieving the synthesis of (+)-cubenene with a titer of 597.3 mg/L in *S. cerevisiae* [91]. Zhao et al. screened three linalool synthases from plants, including *Valeriana officinalis*, *Lippia dulcis* and *Ocimum basilicum*, finding that the linalool synthase from *V. officinalis* was the most active enzyme [92]. Hassan et al. successfully synthesized longifolene in yeast for the first time, with a titer of 36.8 mg/L, by screening three potential longifolene synthases in pine trees [93].

#### 4.1.2. Engineering of Terpene Synthases

It is necessary to modify poorly active monoterpene/sesquiterpene synthases through enzyme engineering. Common enzyme engineering methods include truncation, fusion, site-specific mutagenesis and directed evolution (Figure 2B) [94]. Plant-derived monoterpene synthases are typically localized to specific organelles in plants, such as mitochondria, chloroplasts or the endoplasmic reticulum. These enzymes often contain an *N*-terminal plastid targeting sequence (PTS) in their sequence [14]. When plant-derived terpene synthases are introduced into microbial cells, the cells may not recognize and cleave the signal peptide sequence, resulting in inefficient expression of the terpene synthases. Truncating the *N*-terminus of the PTS is an effective method to increase the activity of monoterpene synthases. For example, the strain expressing truncated linalool synthases showed an 8-fold increase in linalool production compared to the strain expressing with an untruncated enzyme [92]. For the synthesis of myrcene, the truncation of seven myrcene synthases from different sources revealed that trCsMS from *Cannabis sativa* exhibited the best activity [70]. Moreover, the activity of the same monoterpene synthase can be affected by different truncation positions. For instance, Keasling et al. truncated the linalool synthase at two different sites and found that the linalool synthase from *Mentha citrata* with RR site truncation (t67-McLIS) showed the best activity, increasing the linalool production by 17 times compared to that of the untruncated synthase [95]. Jiang et al. performed truncation at four different sites (S14, L28, S43 and S52) of the geraniol synthase (CrGES) derived from *Catharanthus roseus* and identified that the S43-truncated CrGES exhibited the highest activity, with geraniol production reaching 191.61 mg/L, 4.45 times higher than that of the full-length CrGES [96].

Fusion expression of terpene synthases can also effectively enhance their catalytic activity. Fusion expression with short peptide tags is an effective method, as the addition of short peptide tags does not cause steric hindrance and can increase the translation efficiency of the recombinant proteins. Wang et al. fused the linalool synthase with MBP, NusA, GST and the short peptide tag CmR29 and found that only the expression of the CmR29*bLIS fusion protein increased the linalool production, confirming the effect of short peptide tags in enhancing terpene synthase activity [97]. Subsequently, they also fused the linalool synthase with the CmR29 tag, resulting in a linalool titer of 1342.6 mg/L [72]. Zhang et al. fused three variants of CMR29 to the *N*-terminus of the santalene synthase and obtained the highest santalene production of 1078.8 mg/L, which is 131% higher than that of the strain without the tagged synthase [98]. Additionally, the fusion of terpene synthases with GPP/FPP synthases can effectively increase the production of monoterpenes/sesquiterpenes. Ignea et al. fused the sabinene synthase (SpSabS1) from *Salvia pomifera* with ERG20, resulting in a 3.5-fold increase in sabinene production [99]. Albertsen et al. fused the patchoulol synthase with FPP synthase and found that a short linker was more effective in enhancing patchoulol synthase activity [100]. Sarria et al. found that fusion expression of the phellandrene synthase and GPP synthase with a tandem Gly-Ser-Gly (GSG) linker could promote phellandrene production, achieving the highest phellandrene production of 32 mg/L [101]. Recently, Vickers et al. fused the nerolidol synthase with FPP synthase using four different linkers, leading to a 110-fold increase in nerolidol shake-flask production, reaching 4.2 g/L [74]. Moreover, synthetic enzymes can also be co-localized on an artificially created protein scaffold for expression. The synthetic protein scaffold can help intermediates in the metabolic pathway transfer directly from one enzyme to the next, enhancing synthesis efficiency. Tippmann et al. established a terpene synthase activity screening system based on the recognition of affibodies to their anti-idiotypic partners and co-localized the FPP synthase and the farnesene synthase on this scaffold in yeast. By adjusting the ratio of the protein scaffold and the enzyme, they increased farnesene production by 135% [102].

Based on the crystal structure of terpene synthases or structural computational models, the rational or semi-rational design of site-directed mutagenesis is also an effective method to enhance their catalytic efficiency. Residues surrounding the active site and substrate binding site are generally considered to be closely related to the catalytic efficiency and catalytic specificity of the enzyme [103]. Zhang et al. performed a homology modeling analysis of the germacrene A synthase (AvGAS) based on the structure of the 1,8-cineole synthase (PDB ID: 5NX6) and selected 16 specific residues for mutagenesis based on the combination of the structural analysis and the homology sequence analysis. They found that the mutations F23W and F23V significantly increased germacrene A production, by 35.2% and 21.8%, respectively [104]. Shukal et al. established the structure model of the viridiflorol synthase based on the structure of the epi-isozizaene synthase (PDB ID: 4ltz) and 15 highly similar synthase sequences. They designed a small library targeting 16 sites and selected 15 mutations based on the phylogenetic tree. Unfortunately, these mutations led to a decrease in the target product. Subsequently, by randomly mutating 400 clones, they found that the combined V314Y and G227C mutations increased viridiflorol production by 38% [61].

Additionally, site-specific mutagenesis of enzymes can also change the type of catalyzed product. For example, Yoshikuni et al. showed the plasticity of the sesquiterpene synthase and the capacity to interconvert it into novel terpene synthases by engineering the plasticity residues [105]. Zha et al. used AlphaFold2 to predict the structure of the santalene synthase (SanSyn) and found that the F441 residue of SanSyn is a key residue that limits the conformation dynamics of the intermediate. After site-directed mutagenesis of F441 to F441V, the SanSyn was able to simultaneously produce α- and β-santalene [106]. Moreover, Kampranis et al. showed that engineering the active-site residues responsible for product specificity could convert cineole synthase to other monoterpene synthases, as well as sesquiterpene synthases [107]. In fact, site-directed mutagenesis of terpene synthases largely depends on enzyme structure information and the enzyme catalytic mechanism. The results of site-directed mutagenesis often cannot meet the expectations. The directed evolution of enzymes does not depend on the identification of enzyme structure and is also an effective method to enhance catalytic efficiency. However, enzyme-directed evolution will create a large library of enzyme mutations, and establishing an effective screening system is an essential requirement. Furubayashi et al. established a terpene synthase screening system based on the competition of the terpene synthase and the carotenoid synthase for their common substrates. Based on this system, they performed random mutagenesis and screening of the catalytic domains of phellandrene synthase, resulting in a 60% increase in phellandrene production in *E. coli* [46,108]. Recently, our research group achieved the directed evolution of the myrcene synthase by establishing a novel myrcene whole-cell biosensor. As a result, we obtained eight improved myrcene synthase mutants, with the highest one displaying a 1.47-fold increase in catalytic efficiency compared to the parent strain and reaching a myrcene titer of 510.38 mg/L [70].

### 4.2. Optimizing Metabolic Pathways

#### 4.2.1. Optimization of MEP and MVA Pathways

In *E. coli*, the precursors IPP and DMAPP produced through the MEP pathway are in low amounts, resulting in the limited synthesis of terpenes. For example, when introducing farnesene synthase into *E. coli* BL21(DE3) to synthesize farnesene with the MEP pathway, the production of farnesene is only 0.325 mg/L [109]. Similarly, when expressing the sesquiterpene synthases EIZS, PentS and MrTPS2 in *E. coli* DH1, the production values of epi-isozizaene, pentalenene and α-isocomene are only 0.54 mg/L, 0.19 mg/L and 0.01 mg/L, respectively [38]. Increasing the precursor metabolic flux is an effective strategy to improve the production of terpenes (Figure 2B). The rate-limiting steps in the MEP pathway are not well understood, and enzymes such as Dxs, IspD and IspF in the pathway are subject to feedback inhibition [110,111]. Overexpression of these enzymes leads to the accumulation of the intermediate metabolite MECPP (2-C-methyl-D-erythritol-2,4-cyclodiphosphate), which limits the enhancement of terpene production via modification of the MEP pathway [112].

Researchers have shown that introducing the exogenous MVA pathway for increasing the precursor supply is more beneficial than modifying the MEP pathway in *E. coli* [113]. The MVA pathway genes are a requirement, and are commonly derived from *Enterococcus faecalis*, *Saccharomyces cerevisiae*, *Streptomyces* sp. CL190, etc. [114]. For instance, Lee et al. constructed a plasmid containing all genes from the MVA pathway derived from *S. cerevisiae* and achieved a limonene titer of 400 mg/L in shake-flask fermentation using glucose as the carbon source [115]. Subsequently, the limonene production was further enhanced to 3.1 g/L through the optimization of fermentation [45]. Bao et al. separated the *atoB*, *HMGS*, *HMGR*, *MK*, *PMK*, *PMD* and *idi* into two expression cassettes and introduced the resulting plasmid into *E. coli* MG1655, and the production of pinene increased to 104.6 mg/L, which was 13.1 times that of the initial strain [116]. In addition, the production of sesquiterpenes, including caryophyllene, β-eudesmol, farnesol and protoilludane, was also increased through the expression of the exogenous MVA pathway, codon optimization, etc. [117,118,119,120]. Moreover, the synthesis of farnesene (1.1 g/L) expressing the exogenous MVA pathway, FPP synthase and farnesene synthase in *E. coli* was 2000 times than that of strains expressing only farnesene synthase [109].

Typically, exogenous pathway genes are constructed into plasmids and transformed into *E. coli* for the synthesis of targets. However, the genetic instability of plasmids and the metabolic burden imposed by antibiotic expression affect the high-density growth of bacterial cells and product generation. Chromosomal integration effectively addresses these issues [121,122]. For example, in order to overcome the disadvantages of plasmid expression during β-carotene production, *HMG1*-*ERG12* and *mvaS*-*mvaA*-*mavD1* were integrated into the *E. coli* chromosome at the *pflB* and *frdB* sites. By further regulating the expression of *atoB*, *mvas* and *HMG1*, the titer of β-carotene increased 1.51-fold without affecting the cell growth [123]. However, the gene copy number for chromosomal integration is lower than that of plasmid expression, resulting in lower expression levels. To address this issue, exogenous genes are often expressed under the control of strong promoters (e.g., T7), integrated into multiple copy loci in the genome or expressed by increasing the copy numbers of chromosomal integration [122]. Lee et al. used CRISPR-Cas9 to construct the MVA pathway and squalene synthase into three expression cassettes, including T (pKIKO-CmoxB-PgadE-MevT), M (pKIKO-Kan-lacZ-Ptrc-MevB) and B (pKIKO-Kan-rbsAR-LacI-Ptrc-BS-idi-ispA), and integrated the three cassettes into the *rbsAR*, *lacZ* and *poxB* loci in *E. coli*, respectively. The expression levels of the three loci were ranked as B > M > T, resulting in a 5-fold increase in the yield of squalene [124]. Additionally, the accumulation of IPP and FPP in the terpene synthesis pathway is toxic to cells [125]. The dynamic regulation of the exogenous MVA pathway with promoter engineering or inducible control of the promoter is an effective strategy. For example, by regulating the MVA pathway dynamically, epi-isozizaene, pentalenene and α-isocomene achieved titers of 727.9 mg/L, 780.3 mg/L and 77.5 mg/L, respectively, in *E. coli* DH1 [38].

IPP and DMAPP are derived from the MVA pathway in yeast, which has stronger tolerance to cytotoxic intermediates such as MVA and IPP [125]. Studies have shown that HMGR is a rate-limiting enzyme in yeast, and overexpression of HMGR can effectively increase the production of farnesol, nerolidol and epi-cedrol [126,127]. In addition to HMGR, increasing the expression of other genes in the MVA pathway can also enhance the production of sesquiterpenes. For example, the production of farnesol reached 1.68 g/L by expressing mutated farnesyl diphosphate synthase (ERG20), truncated HMGR and isopentenyl diphosphate isomerase (IDI1) with a galactose-inducible promoter [128]. In another study, the dynamic regulation of ERG20 expression was achieved by replacing the ERG20 promoter and controlling the addition of carbon sources, resulting in a 3.4-fold increase in farnesol production [73]. Furthermore, researchers obtained an FPP overexpression chassis named JCR27 by overexpressing all genes in the MVA pathway in *S. cerevisiae* and achieved the synthesis of multiple sesquiterpenes, including (-)-eremophilene, epi-isozizaene, protoilludene, pentalenene and α-farnesene, with the highest yield reaching 38.8 g/L [32,60,129]. Except for *S. cerevisiae*, other yeast species including *Y. lipolytica* and *R. toruloides* are also excellent hosts for synthesizing terpenes. For instance, in *Y. lipolytica*, the yield of limonene reached 917.7 mg/L by expressing GPPS and limonene synthase, downregulating ERG20 and overexpressing tHMG1 and IDI1 [130]. Zhao et al. achieved the efficient synthesis of bisabolene in *Y. lipolytica* by introducing an exogenous MVA pathway and efflux pumps [78]. In *R. toruloides*, high expression of the MVA pathway genes increased the production of bisabolene to 2.6 g/L [50].

#### 4.2.2. Optimization of the Upstream and Downstream Pathways

In microbial hosts, IPP and DMAPP are synthesized from glycerol-3-phosphate (G3P), pyruvic acid and acetyl-CoA, which are produced through the glycolytic pathway. Enhancing the supplement of these precursors is an effective means for improving the production of terpenes in host cells (Figure 2B). For the MEP pathway, the imbalance supply of G3P and pyruvic acid is a major bottleneck. Jung et al. found that downregulating *gapA* in *E. coli* was more effective than overexpressing *ppsA* for balancing G3P and pyruvic acid, and downregulating *gapA* effectively increased the production of lycopene (97% higher than the starting strain) [131]. Researchers found that in *E. coli*, the combination of the Entner–Doudoroff pathway (EDP) and the Hexose Monophosphate Pathway (HMP) was beneficial for precursor generation and energy supply, and activation of the EDP and PPP pathways increased the yield of isoprene by 1.9-fold [132,133].

For the MVA pathway, it is essential to increase the accumulation of intracellular acetyl-CoA for terpene synthesis. Common methods, including strengthening the endogenous acetyl-CoA synthesis pathway or constructing an exogenous acetyl-CoA synthesis pathway, were applied [134]. For example, Kong et al. knocked out the malate synthase encoding gene *MLS1* and the citrate synthase encoding gene *CIT2* in *S. cerevisiae*, leading to a 38% increase in the production of limonene, reaching 889.54 mg/L [135]. Bai et al. increased the production of β-elemene to 707.18 mg/L (2.7-fold increase) in *E. coli* by enhancing the accumulation of acetyl-CoA through knocking out the competing pathways in central carbon metabolism [136]. In *S. cerevisiae*, the expression of exogenous ACL can convert citric acid in the tricarboxylic acid cycle (TCA) into cytoplasmic acetyl-CoA. Keasling et al. found that ACL from *Aspergillus nidulans* showed the best activity in yeast after screening five different sources of ACL [137]. Our research group significantly increased the production of γ-bisabolene in *S. cerevisiae* by expressing ACL from *Yarrowia lipolytica* [56].

In addition to precursor supply, cofactors such as NADPH and ATP are also crucial for the synthesis of terpenes. The consumption of cofactors for the MEP and MVA pathways differs. For the MEP pathway, the synthesis of one molecule of IPP/DMAPP requires the consumption of three molecules of NAD(P)H, one molecule of ATP and one molecule of CTP. For the MVA pathway, the synthesis of one molecule of IPP/DMAPP requires the consumption of two molecules of NADPH and three molecules of ATP. Common methods used to increase intracellular NADPH accumulation include reducing intracellular NADPH consumption or increasing NADPH synthesis. In *S. cerevisiae*, the production of cubebol was increased by 85% by knocking out the NADPH-dependent glutamate dehydrogenase gene *GDH1*, albeit impacting cell growth [138]. Asadollahi et al. found that overexpressing the NADH-dependent glutamate dehydrogenase gene *GDH2* could compensate for the effects on cell growth caused by the absence of *GDH1* [138]. Moreover, overexpressing the NADH kinase *POS5* to increase intracellular NADPH accumulation led to a 1.8-fold increase in patchouli alcohol production in *S. cerevisiae* [139]. In *Rhodobacter capsulatus*, knocking out two NADPH-consuming enzymes, β-hydroxybutyrate synthase and glutamate synthase, led to a 2.15-fold increase in the production of bisabolene (390.3 mg/L) [140]. In *E. coli*, introducing the NADH kinase *POS5* from yeast and knocking out the NADPH-dependent aldehyde reductase *YjgB* led to a 1.1-fold increase in the production of protoilludene (512.7 mg/L) [141]. In addition to NADPH, an adequate ATP supply is also crucial for increasing the production of terpenes. Chen et al. overexpressed the endogenous adenine phosphoribosyltransferase (APRT), resulting in a 9.4% increase in intracellular ATP content, and also improved the yield of α-farnesene by 10.3% [142].

Moreover, the synthesis of terpenes is also influenced by bypass and competing pathways. The inhibition or knock-out of genes in the competing or bypass pathways is an effective method to increase terpene production. The activity of GPPS is an important factor influencing the production of monoterpenes. By optimizing the ribosome binding site of GPPS or introducing GPPS from plants, the production of geraniol was increased by 6 times in *E. coli*, reaching 2.0 g/L [143,144]. Researchers have also developed a strategy based on the N-degron-dependent protein to regulate ERG20 activity in yeast, and successfully increased the production of farnesol and limonene [145]. Additionally, by knocking out the endogenous NADPH-consuming enzymes OYE2 and ATF1, the production of farnesol was increased by 1.7 and 1.6 times, respectively, and the further dynamic regulation of ERG20 increased the farnesol production to 1.69 g/L in *S. cerevisiae* [73]. FPP is the precursor for sesquiterpenes synthesis, which is also a precursor of squalene required for cell membrane synthesis. Therefore, balancing the FPP supply for the synthesis of sesquiterpenes and squalene is crucial for ensuring normal cell growth and a high production of sesquiterpenes. Current methods used to regulate FPP metabolism include both the degradation of squalene synthase (ERG9) and dynamic regulation of ERG9 [44]. Vickers et al. effectively increased the production of valencene by 86% by establishing an endoplasmic-reticulum-associated protein degradation (ERAD) method to reduce intracellular ERG9 protein levels [146]. Additionally, regulating the expression of *ERG9* using the methionine promoter P_MET3_ or the glucose-regulated promoters P_HXT1/2_ could effectively increase the production of sesquiterpenes [147].

### 4.3. Cell-Level Optimization

#### 4.3.1. Subcellular Localization of Terpene Synthesis

In recent years, it has been found that in addition to traditional pathway engineering strategies, the utilization of microbial organelles or cell membranes for the compartmentalized expression of pathway enzymes can significantly increase the production of terpenes (Figure 2C). The confinement of organelle membranes can prevent the entry of competing pathways and avoid the harmful effects of toxic substances such as terpenes and intermediates accumulating in the cell [148]. Peroxisome is an important double-membrane organelle, where fatty acids are oxidatively broken down into acetyl-CoA. Dusséaux et al. localized the MVA pathway and limonene synthase in the peroxisome of *S. cerevisiae*, resulting in a 125-fold increase in the titer of limonene compared to strains expressing the same enzymes in the cytoplasm [149]. Liu et al. simultaneously synthesized α-farnesene in the cytoplasm and peroxisome of *P. pastoris*, achieving a yield of 2.18 g/L α-farnesene, which was 1.3 and 2.1 times higher than the α-farnesene production synthesized only in the peroxisome or cytoplasm, respectively [150]. Researchers localized the α-humulene synthesis pathway in the peroxisome of *Y. lipolytica*, simultaneously increased the supply of ATP and acetyl-CoA, and optimized the copy number of key genes, resulting in an α-humulene titer of 3.2 g/L [151]. Recently, our research group achieved the efficient synthesis of γ-bisabolene in *S. cerevisiae* in both the cytoplasm and peroxisomes by controlling the size, number and degradation of peroxisomes, resulting in a titer of 2.69 g/L γ-bisabolene [56].

In addition, since the content of acetyl-CoA in mitochondria is 20–30 times higher than that in the cytoplasm, mitochondria are also a favorable organelle for the synthesis of terpenes [152]. Farhi et al. fused the valencene synthase CsTPS1 with the mitochondrial targeting signal peptide COX4, resulting in a 3-fold increase in valencene production compared to production in the cytoplasm [153]. Yee et al. localized all the proteins of the linalool synthesis pathway to the mitochondria to utilize the mitochondrial acetyl-CoA for GPP synthesis, avoiding GPP consumption by the cytoplasmic metabolic pathways. Compared to strains producing linalool in the cytoplasm, the yield of linalool synthesized in the mitochondria increased 6-fold [154]. Additionally, researchers also achieved the synthesis of farnesol in *S. cerevisiae* through dual metabolic engineering of the mitochondrial and cytoplasmic MVA pathways, expressing the ERG20 mutants ERG20F96W/N127W and the farnesol synthase from *Cinnamomum osmophloeum* in both the cytoplasm and mitochondria and downregulating endogenous ERG20, resulting in a farnesol titer of 23.45 mg/L with sucrose as the carbon source [155].

#### 4.3.2. Enhancing Product Efflux Efficiency

Due to the limited storage of terpenes inside cells, especially the excessive accumulation of monoterpenes, which have toxic effects on cells, there is a limit to increasing terpene production [156]. A strategy to effectively reduce cell toxicity is to increase the efflux efficiency of products by overexpressing cell transport proteins (Figure 2D). ATP-binding cassette (ABC) transporters are a large class of membrane proteins involved in the selective transport of different compounds. The ABCB or multidrug resistance (MDR) subfamily mainly transports hydrophobic compounds [157]. Demissie et al. knocked out 16 ABC transporters in yeast and complemented them with the (MDR)-type ABC transporter LaABCB1 from lavender, significantly increasing yeast tolerance to geraniol [158]. AcrAB-TolC is a member of the resistance-nodulation-division (RND) family derived from Gram-negative bacteria, consisting of the inner membrane ATP-binding transporter AcrB, the periplasmic space accessory protein AcrA and the outer membrane channel protein TolC [159]. Niu et al. replaced the acrAB promoter with the strong P_37_ promoter, increasing the tolerance of *E. coli* to pinene [47]. Additionally, Shah et al. significantly increased the tolerance of *E. coli* to linalool by overexpressing the global transcriptional regulator MarA, resulting in a 104-fold increase in colony formation efficiency of strains when linalool was added to solid plates, while strains knocking out marA or AcrAB-TolC were extremely sensitive to linalool [160].

In addition to overexpressing cell efflux pumps, in situ product removal (ISPR) strategies have been used in the synthesis of monoterpenes and sesquiterpenes. Brennan et al. tested the addition of ten organic solvents during fermentation to reduce the cytotoxicity of monoterpenes and found that several solvents, such as dioctyl phthalate, dibutyl phthalate, isopropyl myristate and farnesene, effectively increased the minimum inhibitory concentration (MIC) by 100-fold compared to the control without the addition of organic solvents [161]. Furthermore, dodecane, decane and isopropyl myristate can be used in the fermentation process of sesquiterpenes and can effectively increase the production of sesquiterpenes [162].

#### 4.3.3. Cell Mutagenesis and Adaptive Evolution

Adaptive evolution is a powerful tool for improving the industrial production properties of some strains or unraveling the complex physiological mechanisms of certain strains (Figure 2E). *E. coli* was mutated with atmospheric and room-temperature plasma (ARTP); then, the resulting cells were evolved in media containing 0.5%, 1.0%, 1.5% and 2.0% pinene to select evolved strains based on the IPP/FPP sensor, finally resulting in a 31% increase in pinene production [47]. Additionally, by gradually increasing the concentration of sabinene during *E. coli* cultivation, a strain with significantly improved tolerance to sabinene was obtained, and the sabinene production was increased by 8.43 times compared to the original strain [163]. It was reported that cell growth is severely inhibited when the concentration of limonene exceeds 500 mg/L for *Y. lipolytica* [164]. Li et al. identified an unknown protein, YALI0F19492p, which resulted in an 8-fold increase in the production of limonene by comparative transcriptome analysis. Through further adaptive evolution, the strain achieved a 52% increase in limonene production [165]. Godara et al. selected strains with high β-caryophyllene production through several generations of strain evolution using oxidative stress as the selective pressure. The optimal strain achieved a β-caryophyllene titer of 104.7 mg/L, which was 3 times higher than the initial strain. A genome sequencing analysis revealed that a-factor exporter *SET6* mutation was a key factor in the product improvement [166]. Yao et al. obtained a Crabtree-negative strain of *S. cerevisiae* through adaptive evolution, resulting in a 20.5% increase in farnesene production compared to the wild-type strain [167]. In addition, it is possible to enable the host to utilize alternative carbon sources such as xylose and biomass hydrolysate for growth and product synthesis with adaptive evolution [168].

## 5. Conclusions and Outlook

Fuel manufacturing is moving towards a greener direction, and the use of biomass as raw material for fuel synthesis has irreplaceable and significant advantages, leading to sustainable and environmentally friendly fuel production. Terpene-derived fuels, with complex and diverse structures, have great potential to replace traditional HED fuels, ensuring a sustainable and secure supply of HED fuels to a certain extent. While terpenes are widely present in nature, their natural content is insufficient to meet the demands for large-scale fuel production and application. However, with the advancement of synthetic biology and metabolic engineering, engineered microorganisms for fuel synthesis have rapidly progressed. The development of efficient microbial cell factories offers promising prospects for the supply of HED fuels derived from terpenes.

Despite the considerable efforts made in research on monoterpene and sesquiterpene fuels, there are still some challenges that need to be addressed. Firstly, while there is a wide structural diversity of monoterpenes and sesquiterpenes, there are not many reported terpene-derived fuel molecules that have undergone actual performance testing. The nature of fuel is closely related to its molecular structure, and the steric effects often result in significant differences between predicted performance and actual values, particularly in low-temperature performance, limiting their subsequent applications. Therefore, in the future, it is necessary to synthesize more types of terpene fuel molecules, determine their actual performance values and conduct in-depth research on the relationship between the molecular structure and properties of terpene-derived fuels to provide theoretical guidance for synthesizing high-performance fuels. Secondly, the current yields of terpenes in microorganisms, especially monoterpenes, are low, and the cost is still high, making it difficult to meet industrial production levels. Therefore, further research is needed to investigate the key biological elements in the pathway of terpene synthesis, design and reconstruct the metabolic pathways of chassis cells, optimize the synthetic pathway and metabolic network, and enhance fermentation processes and intelligent control strategies to increase the yield and productivity of terpene fuels. Thirdly, the comprehensive performance of some terpene-derived HED fuels is not as good as traditional petroleum-based HED fuels. However, due to the plasticity of terpene structures, their density and low-temperature performance can be improved in the future through condensation, cyclopropanation, introducing side chains or controlling spatial configurations. Additionally, terpene-derived fuels can be used in combination with traditional fuels to enhance the overall performance of the fuel.

## Data Availability

There is no new data were created.

## Abbreviations

EMP: Embden–Meyerhof Pathway; HMP, Hexose Monophosphate Pathway; EDP, Entner–Doudoroff pathway. Pathway intermediates: Ac-CoA, Acetyl-CoA; PYR, pyruvic acid; G3P, glyceraldehyde 3-phosphate; AcAc-CoA, acetoacetyl-CoA; HMG-CoA, 3-hydroxy-3-methyl glutaryl coenzyme A; MVA pathway, mevalonate pathway; MEP pathway, 2-C-methyl-D-erythritol 4-phosphate pathway; MVP, mevalonate-5-phosphate; MVPP, mevalonate-5-pyrophosphate; IPP, isopentenyl diphosphate; DMAPP, dimethylallyl diphosphate; GPP, geranyl pyrophosphate; FPP, farnesyl pyrophosphate; DXP, 1-deoxy-D-xylulose-5-phosphate; MEP, 2-C-methyl-D-erythritol 4-phosphate; CDP-ME, 4-(cytidine 5′-diphoospho)-2-C-methyl-D-erythritol; CDP-ME2P, 2-phospho-4-(cytidine 5′-diphoospho)-2-C-methyl-D-erythritol; MECDP, 2-C-methyl-D-erythritol2,4-cyclodiphosphate; HMBPP, 1-hydroxy-2-methyl-2-butenyl 4-diphosphate. Enzymes: AtoB, acetyl-coA thiolase; HMGS, 3-hydroxy-3-methyl glutaryl coenzyme A synthase; HMGR, 3-hydroxy-3-methyl glutaryl coenzyme A reductase; MK, mevalonate kinase; PMK, phosphomevalonate kinase; PMD, mevalonate 5-pyrophosphate decarboxylase; IDI, isopentenyl pyrophosphate isomerase; DXS, 1-deoxy-D-xylulose 5-phosphate synthase; DXR, 1-deoxy-D-xylulose 5-phosphate reductoisomerase; MCT, 2-C-methyl-D-erythritol-4-phosphate cytidylyltransferase; CMK, 4-(cytidine 5′-diphosphate)-2-c-methyl-D-erythritol kinase; MCS, 2-C-methyl-D-erythritol-2,4-cyclodiphosphate synthase; HDS, 1-hydroxy-2-methyl-2-butyl-4-diphosphate synthetase; HDR, 1-hydroxy-2-methyl-2-butyl-4-diphosphate reductase; MTPS, monoterpene synthase; STPS, sesquiterpene synthase.
